# Dr. J. William White

**Published:** 1916-06

**Authors:** 


### OBITUARY

---

**Readers are requested to report promptly the death of all physicians in Western New York, or former residents of this region, or graduates of any medical school in Western New York, and to notify the families of the deceased of our desire to publish adequate Obituary notices.**

---

**Dr. J. William White,** University of Pennsylvania, 1871, died at his home in Philadelphia, after a long illness, April 24, aged 65, having been born in Philadelphia, Nov. 2, 1850. Immediately after his graduation, he accompanied Prof. Louis Agassiz on the Hessler expedition to the West Indies and South America. He early specialized in surgery and was well known as an author, teacher and surgeon, especially in genito-urinary conditions though not limiting his attention to the latter. He became a member of the faculty of the University of Pennsylvania in 1881, soon afterward becoming Prof. of Genito-urinary Surgery and Professor of Clinical Surgery, in which capacity the editor enjoyed his teaching in 1888-9. In 1900, he was appointed John Rhea Barton Prof. of Surgery and, at the time of his death, was Emeritus Prof. of Surgery and a member of the board of trustees. He was a man of culture and of elegance in manner and dress, balanced by a most kindly and sympathetic nature and by studious habits and unusual practical skill. His brain was left to the Wistar Institute of Anatomy. His body was cremated, without religious services, in consistent respect to his ethical conceptions.

